# Glutamate-Gated NMDA Receptors: Insights into the Function and Signaling in the Kidney

**DOI:** 10.3390/biom10071051

**Published:** 2020-07-15

**Authors:** José M. Valdivielso, Àuria Eritja, Maite Caus, Milica Bozic

**Affiliations:** Vascular and Renal Translational Research Group, Institute for Biomedical Research in Lleida (IRBLleida) and RedInRen Retic, ISCIII, 25196 Lleida, Spain; aeritja@irblleida.cat (À.E.); mcaus@irblleida.cat (M.C.)

**Keywords:** NMDAR, glutamate, receptor, kidney, signaling, renal disease

## Abstract

*N*-Methyl-d-aspartate receptor (NMDAR) is a glutamate-gated ionotropic receptor that intervenes in most of the excitatory synaptic transmission within the central nervous system (CNS). Aside from being broadly distributed in the CNS and having indispensable functions in the brain, NMDAR has predominant roles in many physiological and pathological processes in a wide range of non-neuronal cells and tissues. The present review outlines current knowledge and understanding of the physiological and pathophysiological functions of NMDAR in the kidney, an essential excretory and endocrine organ responsible for the whole-body homeostasis. The review also explores the recent findings regarding signaling pathways involved in NMDAR-mediated responses in the kidney. As established from diverse lines of research reviewed here, basal levels of receptor activation within the kidney are essential for the maintenance of healthy tubular and glomerular function, while a disproportionate activation can lead to a disruption of NMDAR’s downstream signaling pathways and a myriad of pathophysiological consequences.

## 1. Introduction

Glutamate-gated receptors (GluRs) are broadly expressed in the central nervous system (CNS) and have paramount roles in excitatory synaptic transmission and synaptic plasticity [1]. GluRs are divided into two different receptor family subgroups, ionotropic and metabotropic GluRs [2,3]. Based on the pharmacological characteristics of the selective synthetic agonist that binds them, ionotropic receptors can be further classified into three specific types of receptors [1]: *N*-methyl-d-aspartate acid (NMDA), α-amino-3-hydroxy-5-methyl-4-isoxazolepropionate acid (AMPA) and kainate (KA) receptors [2,3,4,5,6]. A fourth class of ionotropic GluRs has also been described and is called δ-receptors. δ-receptors show structural similarity to AMPA and KA receptors; however, their functionality greatly differs from these two types of receptors [3,5,6,7]. The *N*-methyl-d-aspartate receptor (NMDAR) is a glutamate-gated non-selective cation channel [1] with a particular molecular structure and specific pharmacological and functional properties [2,7,8]. One of the fundamental characteristics of NMDAR is that its activation followed by an influx of calcium ions can set off an array of calcium-mediated intracellular events that play crucial roles in various aspects of the physiology of complex organisms [9]. NMDAR performs a multitude of normal physiological functions within the CNS [10,11,12,13]. However, it is also implicated in the alterations observed in various diseases of the brain [14]. Survival of many types of neurons strongly depends on physiological levels of NMDAR activity [12], while complete absence [12] or abnormal expression levels or altered function of this receptor have been implicated in different neurological diseases and pathological conditions [15]. In the last decade, an expanding body of data demonstrated the presence of functional NMDAR in a myriad of non-neuronal cells and tissues, where it plays important roles in various physiological and pathological processes [16,17,18,19,20,21,22,23].

The current review explores contemporary knowledge and understanding of physiological and pathophysiological functions of NMDAR in the kidney and focuses on recent findings related to signaling pathways involved in NMDAR-mediated responses in the kidney.

## 2. NMDA Receptor: Structure, Distribution and Functionality in the Kidney

### 2.1. NMDAR Subunit Composition

**Figure 1:**
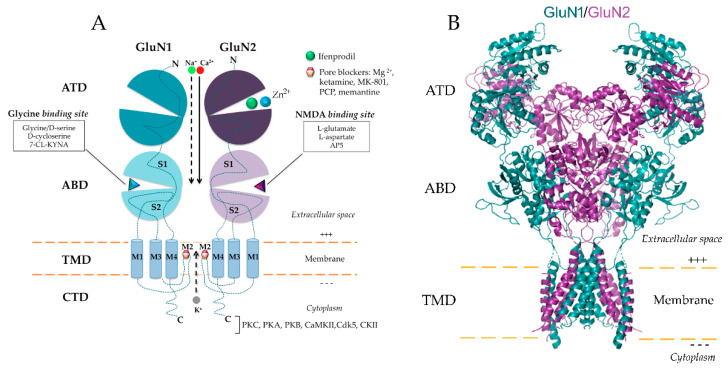
Structure of GluN1/GluN2 *N*-methyl-d-aspartate receptor (NMDAR) with functional domains and ligand-binding sites. (**A**) Schematic diagram of GluN1/GluN2 NMDAR. NMDAR is a heteromeric protein complex that is most often composed of two GluN1 and two GluN2 subunits of the identical or distinctive subtypes. Combinations of GluN1 and GluN2 subunits are indispensable for the composition of the functional non-selective cation channel, which allows influx of Ca^2+^ and Na^+^ ions and efflux of K^+^ ions. High permeability for calcium ions makes NMDAR an important player in diverse physiological and pathological processes. Each subunit of NMDAR is composed of a large extracellular amino-terminal domain (ATD), one agonist-binding domain (ABD), a transmembrane domain (TMD) and carboxyl-terminal domain (CTD). TMD contains three transmembrane helices (M1, M3, M4) and a membrane re-entrant loop (M2). (**B**) Crystal structure of the human GluN1/GluN2 NMDAR in the glutamate/glycine-bound state at pH 7.8 (Protein Data Bank accession no. 6IRA; Zhang et al. 2018 [43]). PKC: protein kinase C; PKA: protein kinase A; PKB: protein kinase B; CaMKII: calmodulin-dependent protein kinase II; Cdk5: cyclin-dependent kinase-5; CKII: casein kinase II; 7-CL-KYNA: 7-Chlorokynurenic acid; AP5: D-2-Amino-5-phosphonopentanoic acid; Mg^2+^: Magnesium; Zn^2+^: Zinc.

NMDAR is a heteromeric protein complex with unique functional properties highly dependable on receptor subunit composition [24]. Functional NMDAR usually requires members from every family of subunits, being mostly found as tetramers composed of two GluN1 and two GluN2 subunits of the identical or distinctive subtypes [7,25,26] (Figure 1). The essential GluN1 and GluN2 are the main subunits of the NMDAR, indispensable for the creation of a functional channel [1,27,28]. The GluN2 subunit family is comprised of four members (GluN2A, -2B, -2C and -2D) [29,30,31] and is involved in the modulatory properties of the receptor, rather than being essential for its function. During the past decade, a novel subunit of the NMDAR family, GluN3, has been discovered and described [32,33]. The GluN3 subunit was found in the form of GluN3A and GluN3B [34] and is able to bind glycine [35]. GluN1 is able to assemble a complex with either GluN3A and/or GluN3B, thereby composing a functional receptor [36,37], where both subunits bind glycine for NMDAR activation. The assembly of such subunits forms a receptor that is not permeable to Ca^2+^ ions and is non-responsive to Mg^2+^, MK-801, memantine and competitive antagonists [38,39]. Furthermore, the GluN3A subunit, when composing an assembly with GluN1 and GluN2, has the capacity to decrease NMDA-evoked currents [40] and calcium permeability of the NMDAR in various cell types [41,42].

### 2.2. Distribution of NMDAR in the Kidney

Besides NMDAR’s wide distribution within the central nervous system, numerous scientific reports have described that functional NMDAR is also expressed in a myriad of non-neuronal cells and tissues such as adrenal glands [44], parathyroid gland [22], lung, thymus, stomach [45], human keratinocytes [17,46], lymphocytes [16], bone cells [18,47], rat cardiomyocytes [46], embryonic [48] and adult heart [45,49,50,51], artery [45,49,52], spleen, ovaries [53], pancreas, skeletal muscle [54], lower urogenital tract [55], renal pelvis [56] and kidney [45,50,57,58], as detailed in a very recent review [23]. Table 1 shows a brief summary of the expression of NMDAR subunits in different regions of the kidney. Results from Leung et al. [45] showed an abundant presence of the GluN1 in kidney medulla and cortex, specifically in the renal proximal tubule. With respect to GluN2 subunits, only GluN2C was measurable in the rat kidney [45]. Deng et al. [50] confirmed the existence of GluN1 in the proximal tubules of the kidney, specifically on its basolateral side. Furthermore, GluN1 and all four GluN2 subunits were shown to be present in proximal tubular kidney (HK-2) cells [57], whereas opossum kidney cells, MDCK and LLC-PK1 cells express both GluN1 and GluN2C [45]. Zhang et al. [59] showed that GluN1 and GluN2A represent the major NMDAR receptor subunits in rat glomeruli, with GluN1 and GluN2A-D subunits detected in glomerular podocytes [60]. Additionally, functional NMDAR was detected in human [61] and mouse [61,62] podocytes. Significant expression of GluN3A and GluN3B was found in the neonatal kidney, while GluN3A subunit showed continued expression in the medulla of the adult mouse kidney [63].

### 2.3. Functional Properties of NMDAR in the Kidney

NMDAR is a complex heterotetrameric assembly with diverse pharmacological properties that can be regulated positively and/or negatively by different exogenous and endogenous compounds. The unique property of NMDAR is reflected in the necessity of simultaneous binding of two agonists, glutamate and glycine, as a prerequisite for the channel activation and its subsequent opening [33,65]. After being produced by the body, endogenous agonists could be transferred to the kidney’s NMDAR through the circulation or released by neural or renal cells. For instance, glutamate (Glu) is produced naturally in the body and is used for a variety of functions on a daily basis. In a normal fasted state, the kidney takes up glutamine from the blood and metabolizes it primarily by the intramitochondrial phosphate-dependent enzyme glutaminase [66], yielding ammonia and glutamate. Glutamate found in the circulation can be further filtered by kidney glomeruli and reabsorbed in the proximal tubule [67]. Under normal healthy conditions, glutamate concentrations found in the plasma range between 10–50 µM [68] and glycine up to 300–400 µM [69]. Although the actual concentration of glutamate reached in the kidney is unknown, renal uptake of glutamine in the human body ranges between 7 and 10 g/day [70]. Besides glutamate and glycine, several other molecules that act as co-agonists of NMDAR were also detected in the circulation, such as l-aspartate between 20 and 30 µM [69], d-serine between 200 and 250 µM and alanine between 500 and 600 µM [69]. Another non-classical agonist of NMDAR, l-homocysteine, has been found in the circulation in a range of 4–6 µM [69] and is known to play a role in the initiation of glomerular injury [59].

It has been demonstrated that extracellular concentrations of various amino acids are highly regulated through excitatory amino acid transporters (EAATs), which have also been detected in the kidney [67,71,72]. Therefore, EATTs play an important role in the regulation of glutamate levels in the kidney, as well as other amino acids. Renal handling of amino acids as well as their plasma levels can be altered in various conditions such as chronic renal disease, metabolic acidosis and hyperhomocysteinemia [73], in which altered NMDAR activation seems to be playing an important role. Thus, increased levels of plasma Glu has been reported in uremic patients on hemodialysis [68], suggesting that glutamate elimination declines in kidney disease. High plasma levels of Glu were also detected in different types of tumors [74], as well as in synovial fluid of patients with arthritis [75].

One of the crucial features of NMDAR is voltage-dependent block by magnesium (Mg^2+^) that happens at resting membrane potentials [33,76]. Thus, when the membrane is depolarized, the channel block disappears and Ca^2+^ entry occurs [77,78]. The low affinity-binding site for Mg^2+^ is deep within the channel and modulated by subunit composition. Hence, NMDAR complexes formed by GluN2A or GluN2B subunits have a higher affinity for Mg^2+^ than those containing GluN2C or GluN2D [79]. Dryer and co-workers describing functional properties of podocyte’s NMDAR pointed to atypical characteristics of NMDAR in these renal cells. Namely, it seems that podocyte’s NMDARs do not respond strongly to l-glutamate, l-aspartate or glycine, even at very high concentrations. Nevertheless, they do so when incubated with NMDA [61]. Furthermore, block of the channel with Mg^2^ can be achieved, but with supraphysiological concentrations (5 mM). Indeed, currents can be achieved at a holding potential of −60 mV with lower concentrations of magnesium (0.8 mM) [61]. These currents did not desensitize, even during the course of quite sustained application of NMDA, which agrees with previous data showing that Mg^2+^ binding enhances NMDAR desensitization while decreasing affinity and open channel probability [80]. However, there is still lack of information on the functionality of NMDAR in the other main renal cell type expressing NMDAR such as tubular cells.

## 3. Distinctive Physiological and Pathophysiological Roles of NMDAR in the Kidney

**Figure 2:**
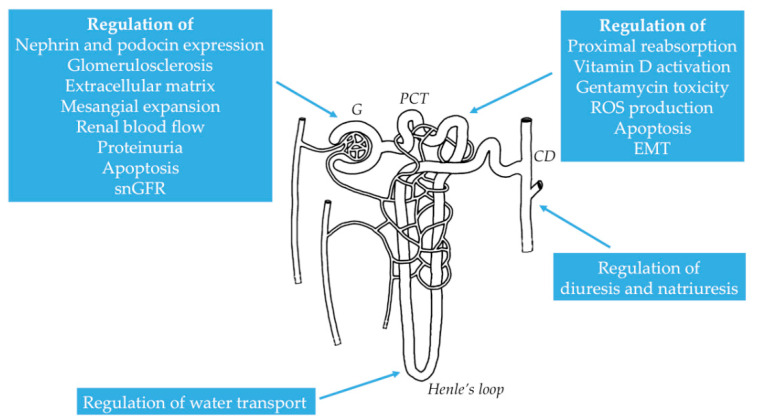
Distinctive roles of NMDAR in various segments of the nephron. G: glomerulus; PCT: proximal convoluted tubule; CD: collecting duct; snGFR: single nephron glomerular filtration rate; EMT: epithelial-mesenchymal transition; ROS: reactive oxygen species.

The kidney is an essential excretory organ that exhibits extraordinary coordination between effective blood flow autoregulation and proximal tubular reabsorption and excretion, as well as glomerular filtration [81], thereby preserving the homeostasis of the body. In the last decade, there has been an increasing body of data demonstrating the role of NMDAR in the glomerular and tubular function (Figure 2). It is evident that basal degree of NMDAR activation is essential for the preservation of normal tubular and glomerular function, while a disproportionate activation can provoke a disruption of renal homeostasis, leading to a diversity of pathophysiological sequels seen as structural and functional organ impairment [23,82].

### 3.1. Physiological Role of NMDAR in Renal Hemodynamics and Glomerular Filtration

Deng et al. [50] were among the first who pointed out an important role of NMDAR in the control of renal vasodilation and the maintenance of the normal renal function. Namely, intraperitoneal administration of antagonist of the NMDAR (MK-801) and/or an inhibitor of glycine binding to NMDAR (5,7-dichlorokynurenic acid) led to renal vasoconstriction and reduced later renal vasodilatory reaction to glycine infusion in Wistar rats. Applying prior renal denervation in their experiments, the authors showed that the described effects were not mediated through increased renal nerve activity [50]. Bądzyńska et al. [83], supporting previous results, demonstrated that administration of glycine led to an increase of cortical renal blood flow (CBF) and total renal blood flow (RBF) in normal and in spontaneously hypertensive rats (SHR), in the absence of systemic changes in blood pressure. Furthermore, application of glycine alone caused diuresis and natriuresis, although the effect was less effective in SHR. The authors suggested a combined systemic administration of glycine and kynurenic acid (KYNA), a noncompetitive antagonist at the glycine site of the NMDAR, as a strategy to decrease body fluid retention and to lower hypertension, without putting in jeopardy renal function. Slomowitz et al. [84] suggested that NMDAR might be accountable for modulating not only tubular but also glomerular response to glycine. The authors showed that low protein diet in rats led to a drop of glycine-induced vasodilation and glomerular filtration rate (GFR), a response that was related with a downregulation of kidney NMDAR protein expression and a significant decrease in proximal tubular reabsorption. Taking into consideration the fact that NMDAR is present in glomerular and tubular cells, Deng et al. [64] proposed that renal NMDAR could autonomously stimulate proximal tubule reabsorption and glomerular filtration. In their study, application of MK-801 (systemically or by direct application to the glomerulus or proximal tubule by microperfusion), produced significant decrease of single nephron glomerular filtration rate (SNGFR) and reduced reabsorption in proximal tubules of Wistar rats. Additionally, inhibition of NMDAR repressed proximal reabsorption independently of the filtered load and decreased SNGFR independently of tubuloglomerular feedback (TGF). The results implied that NMDARs in the hydropenic rat kidney cortex boosted vasodilation and produced a stimulus for proximal tubular reabsorption [64]. The results indicate that NMDAR modulators with tonic vasodilatatory effect on the glomerular microvasculature might be used as a useful therapeutic tool to regulate TGF and glomerular filtration. Another piece of evidence regarding the function of NMDAR the kidney is the paper of Zakrocka et al. [85], where authors demonstrated that angiotensin II type 1 receptor blockers decreased production of KYNA in a dose-dependent manner via enzymatic inhibition of KYNA synthesis, which influenced kidney function [85].

As seen from previously published data, consumption of certain diets could have a potential effect on the functionality of the NMDAR, as well as on kidney function. Interestingly, monosodium glutamate (MSG) has been regularly used worldwide as a flavor enhancer to stimulate suitable food selection in particular cultures [86]. Hence, a report of Mahieu et al. [87] revealed that chronic administration of MSG in rats raised both GFR and tubular reabsorption of Na, K and water, which was followed by an upregulation of GluN1 in the kidney. Blockade of NMDAR with MK-801 significantly reversed all the changes induced by monosodium glutamate. Hence, persistent activation of NMDAR could lead to changes at both renal and systemic levels, such as kidney failure and hypertension.

Renal NMDAR has been also shown to regulate intracellular calcium levels and water reabsorption, most probably through the actions of its GluN3A subunit expressed in inner medullary collecting duct (IMCD) cells [63]. By knocking down the GluN3A in IMCD cells, the authors detected an elevation of basal intracellular Ca^2+^ concentration, lessened cell proliferation, increased apoptosis and decreased water transport in reaction to the addition of vasopressin [63]. Thus, GluN3A may have an important renoprotective role in IMCDs, enabling the principal cells to reabsorb water and, therewith, helping the maintenance of the countercurrent multiplication system [88,89].

### 3.2. Pathological Role of NMDAR in Different Renal Conditions

As reviewed in this article, NMDAR is widely present in the kidney and has a multitude of roles in various renal physiological processes. Nevertheless, over the last decade it has become increasingly evident that NMDAR also has a paramount role in various pathological processes in the kidney (Table 2).

#### 3.2.1. Role of NMDAR in Renal Fibrosis

Results from our group demonstrated an essential role of NMDAR in the maintenance of epithelial phenotype of renal tubular cells and in the modulation of key steps of tubular epithelial–mesenchymal transition (EMT) [58]. On the one hand, knockdown of GluN1 produced marked alterations in epithelial phenotype of HK-2 cells, seen as a decline of E-cadherin and a rise of α-SMA, besides the alterations in cell morphology [58]. On the other hand, TGF-β1-induced EMT in HK-2 cells was decreased by co-treatment with NMDA, an agonist of NMDAR. In vivo, intraperitoneal administration of NMDA markedly suppressed expression of key markers of renal fibrosis in the unilateral ureteral obstruction mouse model, implying that NMDAR might be the target for the suppression of EMT and therapy of related conditions [58].

#### 3.2.2. Role of NMDAR in Secondary Hyperparathyroidism in CKD

Additional research line from our group described the role of NMDAR in the initiation of secondary hyperparathyroidism (2HPT) in chronic kidney disease (CKD). Namely, activation of NMDAR led to a decreased synthesis of 1,25(OH)_2_D_3_ in HK-2 cells in vitro and 1,25(OH)_2_D_3_ levels in the blood in vivo [57]. Consequently, absence of the suppressive effect of vitamin D on the parathyroid gland led to an increase of blood parathyroid hormone levels. Moreover, animals with 5/6 nephrectomy-induced CKD showed higher levels of renal glutamate in comparison with healthy counterparts, implying an overactivation of tubular NMDAR by glutamate as a probable explanation for the downregulation of 1α-hydroxylase, subsequent decline in 1,25(OH)_2_D_3_ synthesis and the onset of 2HPT related with CKD [57].

#### 3.2.3. Role of NMDAR in Acute Kidney Injury

Acute kidney injury (AKI) is a clinical syndrome defined by rapid reduction in kidney function [92], which encompasses both injury and impairment of renal function. One of the main causes of AKI is renal ischemia–reperfusion injury (IRI), which is associated with decreased nutrient reserves and oxygen supply, driving apoptosis and necrotic death of tubular cells and consequent impairment of kidney function [90,91]. Yang et al. [90] demonstrated that unilateral ischemia–reperfusion (IR) in rat kidney led to a decreased GFR response, which was associated with enhanced renal GluN1 protein expression. Furthermore, intrarenal arterial NMDA infusion alone led to a decline of GFR in control and IR kidneys. Administration of AP-5, an NMDAR antagonist, managed to ameliorate IR-induced impairment of glomerular and tubular function in both groups of animals, as well as to effectively abrogate NMDA-induced kidney dysfunction. Pundir et al. [91] showed that different NMDAR antagonists such as ketamine, KYNA and magnesium sulfate diminished IR-induced AKI and lessened oxidative stress, pointing to a favorable effect of the antagonism of diverse allosteric sites of NMDAR against IR-induced AKI [91]. A year later, the same research group confirmed the association of AKI with the activation of NMDAR, showing that glycine administration led to an increase in IR-induced AKI in rats, while KYNA ameliorated AKI. The authors pointed to a glycine-mediated activation of NMDARs as accountable for its renal-damaging effects [92]. Singh et al. [93] also corroborated those results and revealed that pioglitazone, a PPAR-c agonist that reduced IR-induced kidney damage, exerted its safeguarding function by inhibiting NMDAR, as pioglitazone protective effect was diminished by foregoing activation of NMDAR [93]. More evidence to support the claim that NMDAR blockade has a beneficial effect in IR-induced AKI is found in recent papers from Kaur et al. [95] and Singh et al. [94]. Namely, administration of curcumin ameliorated IR-induced AKI in a dose-dependent manner, while application of glutamic acid and spermidine abrogated curcumin-mediated renoprotective effect in rat kidneys [95]. Treatment with estradiol mitigated IR-induced oxidative stress and structural damage in renal tissue, while NMDAR agonists abolished estradiol-mediated renoprotection [94]. Another piece of evidence associating excessive NMDAR signaling with acute kidney failure is a work of Lin et al. [96]. Namely, administration of LPS led to an increased expression of GluN1 in renal tubules and marked damage of renal structures in vivo, which was improved by the NMDAR blocker, MK-801. In vitro, LPS caused cell damage in primary rat proximal tubular cells and cultured tubular cell lines, which was alleviated by MK-801 and downregulation of GluN1 in vitro. Moreover, a study from Cauli et al. [105] showed that in rats with acute liver failure (a lethal condition leading to rapid organ decline), blockade of NMDAR delayed death by improving GFR and, therefore, the clearance of cytotoxic ammonia [105]. Recently, Ying et al. [97] showed that treatment with ligustrazine (LGZ) protected against experimental sepsis-associated AKI in mice, probably via downregulation of renal NMDAR expression and reduction of apoptosis [97]. Hence, it appears understandable that in AKI, an uncontrolled glutamatergic signaling through NMDAR is deleterious to the renal tissue, and NMDAR blockade could be a therapeutic option for the improvement of the kidney function.

#### 3.2.4. Role of NMDAR in Glomerular Disorders

The majority of glomerular disorders are defined by an impairment of the glomerular filtration barrier, where podocyte dysfunction plays an essential role. Rastaldi and co-workers were among the first ones to show an important role for NMDAR activation in the preservation of the stability of the glomerular filtration barrier, while disturbance in glutamatergic signaling in podocytes might lead to proteinuric renal disease [62]. The authors observed that, in cultured podocytes, treatment with MK-801 or norketamine hydrochloride gave rise to a significant podocyte cytoskeleton remodeling and markedly reduced surface expression of podocyte nephrin, while the addition of 50 µM of NMDA for 15 min overturned these changes [62]. Both antagonists showed a direct effect on glomerular filtration, significantly increasing glomerular albumin permeability in isolated rat glomeruli. In vivo, intraperitoneal administration of norketamine in Balb/c mice for 3 days demonstrated a marked elevation of U_Alb_/U_Creat_ accompanied by a reduced expression of glomerular nephrin [62]. However, Anderson et al. [61] showed that sustained activation of NMDAR in podocytes could be highly detrimental for the glomerular filtration, causing the loss of proteins vital for the normal function of slit diaphragms. Namely, 6 h of exposure to 50 μM NMDA led to a marked decrease in nephrin expression in podocytes in vitro. A year later, the same research group provided evidence that more prolonged activation of NMDAR in podocytes (24 h) reduced total and cell surface expression of podocyte markers nephrin and podocin, while 72 h of exposure to NMDA evoked a significant apoptotic podocyte cell death [98]. In line with previous results, Zhang et al. [59] described a role for NMDAR in hyperhomocysteinemia (hHcys)-induced glomerulosclerosis. Namely, rats affected by hyperhomocysteinemia showed a significant increase of GluN1 and GluN2 subunits in their glomeruli, which was abolished by treatment with MK-801. Obtained results corroborated the involvement of NMDAR in the pathogenesis of glomerulosclerosis induced by hHcys [59].

It has also been shown that NMDAR has an important role in the initiation of diabetic nephropathy, more precisely in extracellular matrix (ECM) remodeling [99]. Thus, in Akita mice, Kundu et al. [99] detected an increase of GluN1 in diabetic kidneys and decreased plasma levels of hydrogen sulfide (H_2_S), which was associated with an increased expression of MMP-9, connexin-40 and -43, responsible for an ECM remodeling [99]. In vitro, glomerular endothelial cells exposed to high glucose showed an increase of GluN1 and MMP-9, attenuation of H_2_S production, and dysregulation of connexin expressions, which was normalized after inhibition of NMDAR by MK-801 and H_2_S treatment. The authors suggest that oxidative stress in diabetic kidney lays upstream the upregulation of NMDAR, leading first to an increase of MMP-9 causing downregulation of H_2_S, which in turn induces GluN1 expression. Described results were later corroborated by a follow-up paper of the same group where treatment with H_2_S in vivo ameliorated kidney function in diabetic mice by way of modulation of NMDAR, MMP-9 and connexin pathways [100]. More data to confirm the role of NMDAR in diabetic nephropathy come from Dryer and co-workers, who demonstrated that antagonism of NMDAR reduced development of diabetic nephropathy in Akita mice by decreasing 24 h albumin excretion and mesangial matrix expansion while improving glomerular ultrastructure [60]. Furthermore, downregulation of NMDARs (GluN1-shRNA) and its chemical inhibition with MK-801 significantly alleviated mesangial expansion and proteinuria in 8-month-old chemically induced diabetic mice and 6-month-old db/db mice in vivo, as well as protected podocytes against high glucose injury in vitro [101].

#### 3.2.5. Role of NMDAR in Nephrotoxic Renal Failure

Nephrotoxicity is one of the most prevalent kidney problems that appear when the body is exposed to a toxin or drug that can lead to kidney damage. Silverstein and co-workers [102,103], using in vivo and in vitro approaches, assessed the role of NMDAR in renal cell toxicity. Keeping in mind that NMDAR is expressed in the renal proximal tubule [102] and that it plays an important role in gentamicin ototoxicity [106,107,108] as well as in gentamicin nephrotoxicity [102], the authors postulated that gentamicin could activate NMDAR and might play a role in kidney injury produced by this antibiotic. Namely, treatment of Sprague-Dawley rats with gentamicin (short-term) led to a substantial increase of GluN1 and GluN2C subunits in the renal cortex [102], while exposure to MK-801 attenuated the renal damage, revealing a major role for NMDAR in the gentamicin model of renal toxicity. Leung et al. [103], using MDCK cells and proximal tubule-like opossum kidney cells, additionally showed that immoderate activation of the NMDAR (10 mM glutamate), as well as the excessive blockade of this receptor with MK-801 or CPP led to harmful effects on survival of kidney cells [103]. Recent work from Gao et al. [104] showed that NMDAR activation caused by glyphosate treatment led to increased oxidative stress and apoptotic death of renal epithelial cells, while blockade of NMDAR ameliorated glyphosate-induced cell damage [104].

## 4. NMDAR-Mediated Signaling Pathways in the Kidney

**Figure 3:**
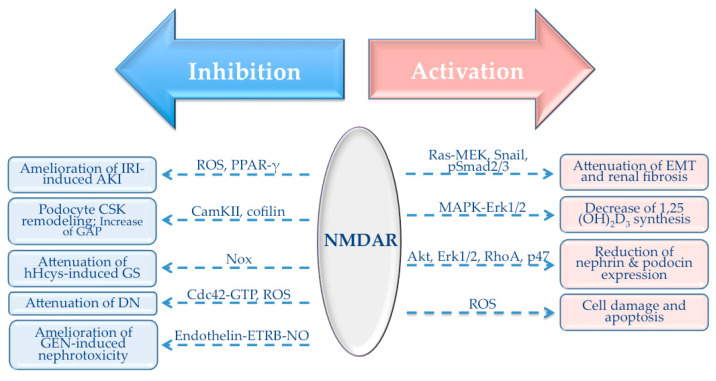
Signaling pathways involved in NMDAR-mediated responses in the kidney. AKI: acute kidney injury; CSK: cytoskeleton; GAP: glomerular albumin permeability; DN: diabetic nephropathy; GEN: gentamicin; EMT: epithelial–mesenchymal transition. Thick arrows refer to the mode of action of the NMDAR (red—activation; blue—inhibition). Intermittent arrows represent signaling pathways involved in a particular process or a disease where NMDAR is involved. For example, activation of NMDAR in the kidney leads to an attenuation of EMT and renal fibrosis with the involvement of Ras-MEK, Snail and pSmad2/3 pathways. On the other hand, inhibition of the NMDAR leads to an amelioration of IRI-induced AKI where ROS and PPAR-γ play a central role.

NMDAR is a cation channel whose activation and consequent influx of Ca^2+^ ions could set off an array of Ca^2+^-mediated signaling events that participate in regulation of distinctive cell functions. NMDAR-mediated signaling processes have been extensively investigated within the CNS; however, experimental data describing signaling responses elicited by peripheral NMDAR activation/inhibition are very scarce. The cornerstone of this section will be an overview of signaling pathways involved in NMDAR-mediated responses in the kidney (Figure 3).

### 4.1. Role of NMDAR in TGF-β1 Signaling Pathway

It has been reported that activation of NMDARs could modulate activity of various signaling molecules in the CNS, such as the MAPK-Erk [1,109,110,111,112], PI3K-Akt [113], small GTPase Ras [111,114] and calmodulin-dependent protein kinase II (CaMKII) [12]. Distinct levels of Ca^2+^ entry through NMDAR could activate stimulatory and/or inhibitory pathways that can govern downstream signaling cascades [110,112]. Results from our laboratory showed that treatment with NMDA decreased TGF-β1-induced phosphorylation of Erk1/2 and Akt and the activation of Ras in HK-2 cells, suggesting that NMDA attenuated TGF-β1-induced EMT of renal epithelial cells by inhibiting the Ras-MEK pathway [58]. Furthermore, co-incubation of HK-2 cells with TGF-β1 and thapsigargin (TG), a non-competitive inhibitor of smooth endoplasmic reticulum Ca^2+^-ATPase (SERCAs) able to raise intracellular calcium in proximal tubular kidney cells in vitro [115], had the opposite effect on the activation of Ras-GTP and phosphorylation of Erk1/2 than NMDA did, pointing to specific effects of Ca^2+^ influx through NMDAR [58]. Moreover, in the absence of Ca^2+^ from the medium, NMDA treatment did not reduce TGF-β1-induced phosphorylation of Erk1/2 and Akt, suggesting again the specific role of Ca^2+^ influx through the activated NMDAR as accountable for NMDA effect. It has been demonstrated that NMDAR-dependent pathways could be spatially separated in neurons and that NMDARs containing preferentially GluN2B subunits mediate Erk1/2 [112] and Ras-Erk1/2 [111] inhibition rather than their activation in neurons. Indeed, in our study, inhibition of the Ras pathway by NMDA supports the subunit-regulatory hypothesis and suggests that Ca^2+^ entry through GluN2B-NMDAR in HK-2 cells might be accountable for the decrease of activated Ras and subsequently Erk and Akt pathways [58]. Our results also showed that treatment of HK-2 cells with NMDA blunted TGF-β1-induced increase of Snail1 and translocation of pSmad2/3 into the nucleus, important markers of tubular EMT and downstream targets of Ras, thereby corroborating the inhibitory effect of NMDA on EMT of RPTECs [58]. Furthermore, blockade of NMDAR with MK-801 in HK-2 cells co-treated with TGF-β1 and NMDA diminished a decrease of Snail1 caused by NMDA, implying that the above-described effects are NMDAR-specific [58]. An additional line of research from our group showed that sustained activation of NMDAR caused phosphorylation of the MAPK-Erk1/2 pathway in the kidney, as well as in the RPTECs in vitro, which was followed by a significant drop of active vitamin D synthesis. Treatment of HK-2 cells with UO126, the MAPK-Erk1/2 kinase inhibitor, blunted both the activation of the MAPK-Erk1/2 pathway and a decrease of 1α-hydroxylase levels [57]. An effect of NMDA treatment on phosphorylation of Erk1/2 and Akt, as well as activation of small GTPases was also described in podocytes [61,98]. Namely, application of NMDA to podocytes in culture (10 μM) for 2 h caused phosphorylation of Erk1/2 and Akt, suggesting the activation of both signaling pathways. Treatment with 10 μM NMDA did not induce activation of the small GTPase RhoA, although RhoA was markedly activated by exposure to higher concentrations of NMDA, as measured by GST pull-down assay [61,98]. NMDA treatment did not affect levels of total RhoA, and neither significantly changed levels of activated or total Rac1 GTPase in podocytes [98]. Interestingly, pretreatment of podocytes in culture with MK-801 successfully attenuated activation of Erk1/2, Akt and RhoA, indicating that they were governed by the activation of NMDAR [61]. Shen et al. [101] demonstrated that NMDAR had a vital role in the activation of the Rho-like GTPase Cdc42. Namely, downregulation of GluN1 in podocytes in culture and in glomeruli led to a marked increase of Cdc42-GTP levels, with no changes in levels of RhoA and Rac1, after in vitro high-glucose exposure or in vivo induction of diabetes, supporting a direct inhibitory role of NMDARs on Cdc42 activation [101]. Another piece of evidence on the role of NMDAR in the regulation of signaling molecules involved in the maintenance of the cytoskeletal homeostasis is the paper from Giardino et al. [62]. Namely, incubation of podocytes in culture with an NMDAR antagonist induced a rapid decrease in phosphorylation of CaMKII, which subsequently led to a reduction of phosphorylation of the CaMKII downstream target cofilin, implying their possible involvement in actin cytoskeleton remodeling in podocytes [62].

### 4.2. Role of NMDAR in Antioxidant Response

Maintenance of the physiological balance between antioxidants and reactive oxygen species (ROS) is of paramount importance for the normal kidney function. Increase of oxidative stress with subsequent disruption of the normal cellular signaling mechanisms could be an essential trigger for the development of kidney injury. It has been reported that in the CNS, activation of NMDAR could lead to the production of reactive oxygen species [116,117], which subsequently leads to neuronal injury. Similar to findings within the CNS, generation of ROS in the kidney is associated with the activation of peripheral NMDAR in various pathological conditions [91,92,93,94,95,99,100].

Renal ischemia–reperfusion injury (IRI) is associated with activation of NMDAR, generation of ROS and subsequent tissue damage. Pundir et al. [91] demonstrated marked oxidative stress in IRI, seen as elevated MPO, TBARS, SAG and reduced GSH and catalase, which was aggravated upon NMDAR activation and attenuated after NMDAR antagonism. The results were confirmed a year later in a follow-up paper of the same group [92]. Singh et al. [93] showed that pioglitazone ameliorated oxidative stress in renal tissue after IRI by decreasing MPO, TBARS and SAG and increasing GSH through antagonizing NMDAR, thereby protecting kidney against IRI-induced renal damage. The pioglitazone-mediated antioxidant effect in the kidney was attenuated by pretreatment with glutamic acid and spermidine, pointing to a crosstalk between NMDAR and PPAR-γ in renal tissue [93], as previously demonstrated in neuronal tissues [118,119,120]. Similar effects of pharmacological interventions at NMDARs in the kidney are recent papers from Kaur et al. [95] and Singh et al. [94]. Namely, administration of curcumin [95] or estradiol [94] to rats ameliorated IRI-induced increase of SAG, MPO, TBAPS and decrease of GSH in renal tissue, while NMDAR antagonism in terms of pretreatment with spermidine and glutamic acid prevented curcumin- [95] and estradiol- [94] mediated oxidative stress protection. In podocytes, Kim et al. [98] demonstrated that NMDA treatment increased generation of ROS and mobilization of p47^(phox)^ in podocyte culture in vitro, while the latter effect was completely blocked by antagonist MK-801 [98]. These results imply that NMDAR activation in podocytes results in oxidative stress that is partially mediated by modulation of NADPH oxidase [98].

Oxidative stress has a paramount role in the onset and progression of diabetic kidney disease. Kundu et al. [99] demonstrated that reduced production of an antioxidant H_2_S in diabetes triggered a series of downstream cascades involving upregulation of GluN1 and connexins, leading to a decline of renal function. Supplementation of H_2_S managed to prevent increased expression of GluN1 and connexins, ameliorating kidney dysfunction [100]. The results from Kundu et al. [100] are in agreement with the report of Szaroma et al. [121], who demonstrated that administration of NMDA compromised the antioxidant status by decreasing the activity of superoxide dismutase, catalase and glutathione peroxidase and the amount of reduced glutathione in the kidney, leading to an enhanced oxidative stress. Inhibition of NMDAR by MK-801 significantly reduced glomerular Nox activity, Nox-dependent O_2_ production and lipid peroxidation induced by hyperhomocysteinemia (hHcys) in rat kidneys, suggesting an important role of the NMDAR in activation of Nox system in the kidney during hHcys and in the development of hHcys-induced glomerulosclerosis [59]. Furthermore, blockade of NMDAR attenuated glyphosate-induced increase of Ca^2+^ influx and reduced activity of major endogenous antioxidant enzymes such as SOD, CAT and GSH-Px in HK-2 cells, strongly suggesting that activation of NMDAR, accompanied by Ca^2+^ influx and oxidative stress, is involved in glyphosate-induced renal proximal tubule epithelium apoptosis [104]. Treatment of Sprague-Dawley rats with gentamicin led to an increase of endothelin type B receptor (ETBR) in the renal cortex and urinary nitrite concentration, while MK-801 managed to normalize the levels of urinary nitrites [102]. The authors pointed to an activation of entothelin-ETRB-nitric oxide cellular pathway as responsible for NMDAR-mediated kidney cell damage after short-term gentamicin [102].

## 5. Conclusions

Over the past two decades, various studies reported the presence of NMDAR in the kidney and its versatile roles in different physiological and pathological processes. It seems clear that NMDAR has important roles in proximal tubular reabsorption and glomerular filtration, as well as in the preservation of the epithelial phenotype of renal tubular epithelial cells. Its activation is of vital importance for the stability of the glomerular filtration barrier. Nevertheless, it is becoming clearly evident that NMDAR has an important role in the onset of different pathological conditions of the kidney such as diabetic nephropathy and acute kidney injury. It seems evident that both modes of action of the NMDAR, stimulation and inhibition, could have various (favorable/detrimental) effects on the kidney. Namely, on the one hand, activation of the receptor increases renal blood flow and ameliorates tubulointerstitial fibrosis in vivo, while antagonizing this receptor in the kidney ameliorates ischemia-reperfusion-induced AKI and diabetic nephropathy. However, the inhibition of NMDAR in the kidney does not always lead to a favorable effect, nor does activation. That is, inhibition of NMDAR in podocytes causes a disruption of the glomerular filtration barrier, while its hyperactivation could induce apoptosis and reduce levels of active vitamin D, which could give rise to a frequent complication in CKD patients, the 2HPT. In spite of clear evidence that various agonists and antagonists have specific effects on kidney cells in vitro, one should not forsake the fact that certain studies investigating the role of NMDAR in the kidney use intraperitoneal administration of NMDAR agonists/antagonists that could also have actions on NMDAR in other organ systems, including CNS.

It seems clear that each of the above-described functions of NMDAR bears an important therapeutic possibility for the management of various renal conditions. In order to properly understand these functions, as well as to develop useful clinical interventions for various renal pathologies based on NMDAR modulation, we would need (1) extensive elucidation of the functional difference of various NMDAR modulators, as well as (2) development of new ones with selective activity. This strategy will enable us to deduce whether NMDAR comprises prospective targets in the kidney and whether NMDAR inhibitors or activators would be required to positively influence the outcome of the disease.

## Figures and Tables

**Table 1:** Synopsis of the expression of various subunits of the NMDAR in different regions of the kidney. HK-2, human proximal tubular cells; OKs, opossum kidney cells; MDCKs, Madin-Darby canine kidney cells; IMCDs, inner medullary collecting duct cells; LLC-PK1, pig kidney epithelial cells.

NMDAR Subunit	Tissue/Cell Type	Reference
GluN1	HK-2, Kidney (medulla, cortex), tubules, glomeruli, podocytes, OKs, MDCKs, IMCDs, LLC-PK1	[45,57,58,59,60,61,62,63,64]
GluN2A	HK-2, Glomeruli, podocytes	[57,59,60]
GluN2B	Kidney cortex, HK-2, podocytes	[57,58,60]
GluN2C	Kidney (medulla, cortex), MDCKs, HK-2, OKs, IMCDs, LLC-PK1, podocytes	[45,57,58,60,63]
GluN2D	Kidney cortex, HK-2, podocytes	[57,58,60]
GluN3A	Kidney, IMCDs	[63]
GluN3B	Kidney, IMCDs	[63]

**Table 2:** Overview of distinctive physiological and pathophysiological functions of renal NMDARs with signaling pathways involved.

Pharmacological Modulator	Mode of Action	Relation Function/Disease	Signaling Pathway	Reference
MK-801, 5,7-DCKA	Inhibition	Renal vasoconstriction		[50]
Glycine	Activation	Increased RBF, diuresis, natriuresis		[83]
KYNA	Inhibition	Antihypertensive action		[83]
MK-801	Inhibition	Reduction of SNGFR		[64]
MSG	Activation	Increase of GFR and tubular reabsorption of Na, K		[87]
NMDA	Activation	Attenuation of EMT and renal fibrosis	Ras-MEK, Snail, pSmad2/3	[58]
NMDA	Activation	Decrease of 1,25(OH)_2_D_3_ synthesis	MAPK-Erk1/2	[57]
NMDA	Activation	Decrease of GFR		[90]
D-AP5, KYNA, KET, MgSO_4_, PGZ, CUR, E2	Inhibition	Amelioration of IRI-induced AKI	ROS, PPAR-γ	[90,91,92,93,94,95]
MK-801	Inhibition	Attenuation of LPS-induced endotoxemia		[96]
Ligustrazine	Inhibition	Attenuation of experimental sepsis-associated AKI		[97]
Nor-KA, MK-801	Inhibition	Podocyte CSK remodeling; Increase of GAP	CaMKII, cofilin	[62]
NMDA	Activation	Reduction of nephrin and podocin expression; apoptosis	Akt, Erk1/2, RhoA, ROS, p47	[61,98]
MK-801	Inhibition	Attenuation of hHcys-induced GS	Nox	[59]
MK-801, H_2_S, memantine	Inhibition	Attenuation of DN	Cdc42-GTP, ROS	[60,99,100,101]
MK-801	Inhibition	Amelioration of GEN-induced nephrotoxicity	Endothelin-ETRB-NO	[102]
Glutamate/MK-801, GLY	Activation/Inhibition	Cell damage and apoptosis	ROS	[103,104]

D-AP5: D-2-Amino-5-phosphonopentanoic acid; 5,7-DCKA: 5,7-Dichlorokyneurenic acid; KYNA: kynurenic acid (KYNA); KET: ketamine; RBF: renal blood flow; SNGFR: single nephron glomerular filtration rate; EMT: epithelial-mesenchymal transition; MSG: monosodium glutamate; AKI: acute kidney injury; IRI: ischemia-reperfusion injury; CUR: curcumin; E2: estradiol, PGZ: pioglitazone; MgSO_4_: magnesium sulfate; LPS: lipopolysaccharide; nor-KA: norketamine hydrochloride; GS: glomerulosclerosis; GAP: glomerular albumin permeability; CSK: cytoskeleton; DN: diabetic nephropathy; ROS: reactive oxygen species; GEN: gentamicin; NO: nitric oxide; GLY: glyphosate.
